# Mobile learning resources would be an effective way of helping healthcare professionals learn about testicular torsion

**DOI:** 10.11604/pamj.2013.14.115.2624

**Published:** 2013-03-23

**Authors:** Kieran Walsh

**Affiliations:** 1 BMJ Learning, BMA House, Tavistock Square, London WC1H 9JR, UK

**Keywords:** Medical education, medical learning, mobile learning, testicular torsion, healthcare professionals

## To the editors of the Pan African Medical Journal

Dear Editor,

Baruga and Guyton Munabi should be praised for sharing the lessons learned from their case series on testicular torsion [1]. They present an educational emergency - the next question that we should ask is how the educational emergency should be addressed.

In any medical educational project (be it an emergency or otherwise), the first step should be to undertake a needs analysis of the learners and to review the goals of the education. In this case the needs and goals are reasonably straightforward - predominantly frontline healthcare workers need basic education on how to recognise and refer patients with suspected testicular torsion. How should we then satisfy those needs? In this case there is strong reason to believe that the most effective approach would be simple online learning resources freely accessible via mobile technology. Mobile technology is becoming ubiquitous and text based learning resources that are easily translatable into multiple languages can be delivered via this method [2]. Images (which would be particularly suited to the topic of testicular torsion) can also be delivered via this mechanism - as can video. Content can also be made interactive and problem-based or cased-based in order to better engage the learners. When there is a change to the evidence base on how to manage testicular torsion, content can be easily updated. By its nature content delivered via mobile technology will be accessible to all interdisciplinary members of the healthcare team.

Baruga and Guyton Munabi have presented the evidence base - but evidence-based medicine must be more than an academic exercise - the next step is to put the evidence into practice and it would be wise to look at new delivery mechanisms to enable this to happen.

Yours Sincerely, Dr Kieran Walsh

## Conclusion

Simple learning resources delivered via the internet would be an effective way of helping healthcare professionals learn about testicular torsion.
